# Privacy preserving dynamic data release against synonymous linkage based on microaggregation

**DOI:** 10.1038/s41598-022-06182-y

**Published:** 2022-02-11

**Authors:** Yan Yan, Anselme Herman Eyeleko, Adnan Mahmood, Jing Li, Zhuoyue Dong, Fei Xu

**Affiliations:** 1 School of Computer and Communication, Lanzhou University of Technology, Lanzhou, 730050 China; 2 School of Computing, Faculty of Science and Engineering, Macquarie University, Sydney, NSW 2109 Australia

**Keywords:** Computer science, Information technology

## Abstract

The rapid development of the mobile Internet coupled with the widespread use of intelligent terminals have intensified the digitization of personal information and accelerated the evolution of the era of big data. The sharing and publishing of various big data brings convenience and also increases the risk of personal privacy leakage. In order to reduce users’ privacy leakage that may be caused by data release, many privacy preserving data publishing methods have been proposed by scientists in both academia and industry in the recent years. However, non-numerical sensitive information has natural semantic relevance, and therefore, synonymous linkages may still exist and cause serious privacy disclosures in privacy protection methods based on an anonymous model. To address this issue, this paper proposes a privacy preserving dynamic data publishing method based on microaggregation. A series of indicators are accordingly designed to evaluate the synonymous linkages between the non-numerical sensitive values which in turn facilitate in improving the clustering effect of the microaggregation anonymous method. The dynamic update program is introduced into the proposed microaggregation method to realize the dynamic release and update of data. Experimental analysis suggests that the proposed method provides better privacy protection effect and availability of published data in contrast to the state-of-the-art methods.

## Introduction

Big data is rich in sources, diverse in variety, large in volume,and dynamically updated, and therefore, it contains immense value and information. Big data mining and analysis can be applied to social demographic surveys, public health research, transportation planning, social public opinion analysis, business model surveys and adjustments, agricultural output prediction, biological information analysis, and many other areas of technological innovation and application. Therefore, it has recently attracted the attention of governments, industries, and research departments around the world^1–3^. However, big data is a double-edged sword. Big data publishing without reasonable privacy protection is likely to cause the leakage of sensitive information which may endanger the personal safety of users and of their property, affect personal reputation and physical and mental health, or lead to discriminatory treatments^4–6^.

Traditionally, privacy preserving data publishing was realized by deleting identifying attributes that can uniquely identify an individual. Sweeney and Samarati proved that the data subsequent to deleting of the identity attribute may still disclose an individual’s privacy through linking attacks. Therefore, they proposed the *K*-anonymity privacy protection model^7,8^ to deal with privacy leakage. Subsequent theoretical and practical results suggest that although the *K*-anonymity privacy model cuts off the connection between an individual and his/her record in the published data, there still exists some connections between an individual and his/her sensitive information. For instance, if all the records within the same equivalent class have the same or similar sensitive values, the attacker will directly obtain the sensitive information of all the individuals in the same equivalent class. The *l*-diversity privacy protection model^10^ proposed by Machanavajjhala *et al.* requires that each equivalent class contains at least *l* different sensitive values, thereby reducing the ability of the malicious attackers to infer sensitive information. However, non-numerical sensitive information has natural semantic relevance and it is,therefore, difficult to avoid synonymous linkage through different sensitive values. For example, Table 1 depicts a micro patient table with quasi-identifiers {*Age*, *Sex*, *Zipcode*} and sensitive attribute {*Disease*}. Table 2 is a 3-anonymous edition of Table 1 since each group contains at least 3 different records. If an attacker is able to determine that Wilson belongs to group 2 through some background knowledge, then he/she can definitely infer that Wilson has blood disease. Primarily since “anemia”, “leukemia”, and “lymphoma” all belong to blood disease.

Besides, big data publishing is not set in stone. Compared with periodic static data publishing, i.e., census, industry reports, and health statistics (wherein only one centralized release is performed in each cycle and the released data is not added/deleted/modified during the publishing period until the next data release cycle), many big data publishing scenarios are characterized in terms of dynamic changes and real-time updates. For instance, in case of location and trajectory information, real-time traffic and weather forecasts, and epidemic monitoring, the data should be updated/deleted/modified with different time intervals or frequencies. The dynamic release and update of big data pose new challenges to privacy preserving data publishing. It may break the privacy protection model built on the published data and increase the complexity of the implementation of anonymous models. In addition, it may aggravate the relevance of published data and external knowledge, degrade the effect of the privacy protection model, and even lead to disclosure of privacy.

In order to prevent this kind of privacy leakage caused by synonymous linkage of sensitive values, this paper proposed a privacy preserving dynamic data release algorithm based on microaggregation. Our principal contributions are as follows: (1) A series of indicators are designed to evaluate the synonymous linkages between the non-numerical sensitive values in turn facilitating an improvement in the clustering effect of the proposed microaggregation anonymous method; (2) The improved microaggregation algorithm is proposed to enhance the privacy protection effect of the published data by minimizing the distance between records and the total number of linkages, and for maximizing an increase of entropy; (3) The dynamic update program is introduced into the proposed microaggregation method to realize the dynamic release and update of data.

**Table 1:** A micro patient table.

Name	Age	Sex	Zipcode	Disease
Alice	58	F	10030	Anemia
Peter	49	M	10037	Enteritis
Wilson	49	M	10022	Anemia
Jim	51	M	10029	Lymphoma
Rose	50	F	10033	Leukemia
Jenny	33	F	10019	Enteritis
John	36	M	10013	Bronchitis
Karine	37	F	10010	Anemia
Bob	51	M	10024	Leukemia

**Table 2:** The 3-anonymous edition of Table 1.

GID	Age	Sex	Zipcode	Disease
1	[33–37]	*	1001*	Anemia
1	[33–37]	*	1001*	Enteritis
1	[33–37]	*	1001*	Bronchitis
2	[49–51]	M	1002*	Anemia
2	[49–51]	M	1002*	Leukemia
2	[49–51]	M	1002*	Lymphoma
3	[49–58]	*	1003*	Anemia
3	[49–58]	*	1003*	Leukemia
3	[49–58]	*	1003*	Enteritis

The rest of the paper is organized as follows. Section 2 provides an overview of some related studies pertinent to privacy preserving data publishing methods for static and dynamic datasets. Section 3 introduces the basic indicators used in the traditional microaggregation method. Section 4 depicts the salient ideas and design indicators of our proposed algorithm. The proposed dynamic data release algorithm against synonymous linkage (DRASL) has been put forward in Section 5. Section 6 depicts the experimental results, whereas, Section 7 concludes our paper.

## Related Work

Over the past few years, privacy preserving data publishing technology has aroused widespread attention of researchers and achieved many results. Among them, the *K*-anonymity privacy protection model is the most widely used one. The core idea of the *K*-anonymity model is to express the precise value of the quasi-identifier (QI) attribute in a generalized form^9^. The quasi-identifiers are defined as the attributes that cannot directly identify a unique individual, but is sufficiently relevant and can be combined with other attributes to identify a specific individual. The records in the original data table can be segregated into multiple equivalent classes by generalizing the exact value of the quasi-identifiers into a certain value range. Each equivalent class contains at least *K* ($K\ge 2$) records with the same quasi-identifier values, and a certain individual represented by a record cannot be distinguished from other $(K-1)$ records, so as to achieve the purpose of privacy protection. The *l*-diversity^10^, *t*-closeness^11^, and many other improved anonymous models^12–14^ enhanced the privacy protection capabilities of the traditional *K*-anonymity model, and are widely used in the privacy protection of static data publishing. However, most of the data publishing methods based on the *l*-diversity anonymous model adopt the generalization operation on quasi-identifiers. The implementation process of generalization requires large computational cost and leads to significant decrease on the availability of published data.

Actually, the partition of equivalent class based on quasi-identifiers can also be achieved through microaggregation and clustering methods. The original records are partitioned into groups, wherein records in the same group are similar to each other in terms of their quasi-identifiers and each group contains at least *K* records. J. Domingo-Ferrer *et al.* used microaggregation to achieve *K*-anonymity for continuous, ordinal, and nominal data^15^. They also discussed the optimal solutions for the univariate and multivariate microaggregation problems and proposed hierarchical clustering and genetic algorithms to preserve natural data aggregates^16^ which abandons traditional projecting and ranking methods for multivariate datasets and reduces information loss as compared to conventional multivariate microaggregation methods. Most of the existing anonymization techniques neglect nominal semantics in the records, thereby, resulting in negative affects on the utility of anonymization results. In order to solve this issue, Domingo-Ferrer *et al.* proposed a knowledge-based numerical mapping method for nominal attributes and a distance measurement method between records^17^, which facilitates in capturing and quantifying the underlying semantics. The authors further proposed the novel steered microaggregation which can be used to achieve *K*-anonymity, *l*-diversity, *t*-closeness, and differential privacy in the context of static data sets^18^. By controlling tuple reordering, this new concept of microaggregation can also be used to achieve *K*-anonymity on data streams. Shi *et al.* suggested to use distance metrics and information entropy to aggregate data into equivalent groups, thereby, ensuring the protection of individual privacy while minimizing information loss^19^. B. Abidi *et al.* introduced a microaggregation method based on fuzzy possibilistic clustering^21^ which proposes to study the distribution of confidential attributes within each sub-dataset and the privacy parameter *K* is determined by preserving the diversity of confidential attributes within the anonymized microdata. The microaggregation method proposed by Rodríguez-Hoyos *et al.* employed linear discriminant analysis to build microcells^22^. They also proposed several strategies to simplify the distance calculations and element sorting operations for data microaggregation^23^. Pallarès *et al.* proposed an optimized prepartitioning strategy to reduce the running time of *K*-anonymous microaggregation on large datasets^24^.

Lin *et al.* proposed an efficient clustering method for *K*-anonymization which segregates all the records into different subsets and adjust each of the subset to ensure that it contains no less than *K* records^25^. W. Zheng *et al.* suggested to optimize the clustering process by considering the overall distribution of quasi-identifier groups in a multidimensional space^26^. S. Zouinina *et al.* managed to achieve *K*-anonymity by using topological collaborative clustering^27^. Yan *et al.* proposed a weighted *K*-member clustering algorithm which designed a series of weight indicators and enhanced the clustering effect and reduced the unnecessary computation during clustering^28^. Ceccarello *et al.* discussed the *K*-center clustering approach on MapReduce and Streaming platform^29^. Mehta *et al.* put forward an improved scalable *l*-diversity approach to achieve *K*-anonymization for big data^30^. Experimental tests conducted on the distributed programming framework MapReduce demonstrated improvements in running time and information loss. Avara *et al.* proposed a method to protect the privacy of data maintained in cloud^31^, wherein records are clustered using an adaptive *K*-anonymity algorithm. Mehta *et al.* converted the unstructured data to structured form and proposed an improved scalable *K*-anonymization method to achieve privacy preserving unstructured big data publishing^32^. Siddula *et al.* suggested an enhanced equi-cardinal clustering to achieve *K*-anonymity and facilitate node, edge, and attribute privacy for the social network^33^. Navid *et al.* envisaged to protect users’ privacy through the anonymization of social network graphs^34^. The proposed method optimized the clustering process of the *K*-anonymity method by means of the particle swarm optimization algorithm.

## Prior knowledge

In order to facilitate the understanding of subsequent definitions and descriptions, we first provide a unified explanation of the mathematical notations defined and employed in this paper (as depicted in Table 3).

**Table 3:** Description of mathematical notations.

Symbol	Description
*QIA*	Quasi-identifier attributes
$SA_r$	A sensitive attribute value in a record *r*
$SA_{G}$	A set of sensitive attribute values in a group *G*
$D_{SA}\_random$	A random sensitive attribute value $\in D_{SA}$
*GID*	An equivalent group of table *T*
$C_i$	The $i$th continuous attribute $(i = 1,\dots , m)$
*r*	A record included in table *T*
*r*[*QID*]	The value of *r* in quasi-identifier*QID*
$d_C(v_i,v_j)$	Distance between continuous values $v_i$ and $v_j$
$N_s{_j}$	The $j$th semantic correlation nominal attribute $(j = 1,\dots , n)$
$Tree_{N_s}$	Taxonomy tree defined for semantic correlation nominal attribute $N_s$
$|Tree_{N_s}|$	The total number of leaf nodes for $Tree_{N_s}$
$Parent(v_i,v_j)$	A common parent node of $v_i$ and $v_j$ according to their taxonomy tree
$|Parent(v_i,v_j)|$	The total number of leaf nodes with the root $Parent(v_i,v_j)$
$d_{N_s}(v_i,v_j)$	Semantic correlation nominal attribute values $v_i$ and $v_j$
$N_g$	The $g{th}$ non-semantic correlation nominal attribute $(g = 1,\dots , x)$
*p*	The total number of non-semantic correlation nominal attributes $N_g$
$match(v_i,v_j)$	The number of matches between $v_i$ and $v_j$ for attribute *N*
$d_{N}(v_i,v_j)$	Distance between non-semantic correlation nominal values $v_i$ and $v_j$
$O_h$	The $h{th}$ ordinal attribute $(h = 1,\dots , y)$
|*O*|	The number of distinct values in ordinal attribute *O*
$\phi (v)$	The normalize ranking of ordinal value *v*
$rank(\cdot )$	The ranking function
$d_O(v_i,v_j)$	Distance between ordinal values $v_i$ and $v_j$
$d(r_1,r_2)$	Distance between records $r_1$ and $r_2$
*H*(*GID*)	The information entropy of an equivalent group *GID*)
$GID'$	The union of equivalent group *GID* with an added record *r* from table *T*
$\widehat{H}(GID,GID')$	The entropy increase between equivalent groups *GID* and $GID'$
$\mu _{GID}$	The centroid of the equivalent group *GID*
$f(GID,GID')$	The microaggregation clustering metric between *GID* and $GID'$
$|GID_j|$	The number of records in an equivalent group $GID_j$
$|GID_j(v)|$	The number of records $\in GID_j$ with sensitive value *v*
$\gamma (v_i,v_j)$	The common linked value between $v_i$ and $v_j$ of sensitive attribute *SA*
$Link_{SA}(v_i,v_j)$	The number of synonymous linkages between $v_i$ and $v_j$ of sensitive attribute *SA*
*U*	The strictly upper triangular matrix that contains all set of values $(v_i,v_j)_{i\ne j} \in (v_1,\cdots ,v_n)$
$Tlink_{SA}(v_1,\cdots ,v_n)$	The total number of synonymous linkages in the set $(v_1,\cdots ,v_n)$
$Pr_{SA}(v_1,\cdots ,v_n)$	The probability mass synonymous linkage of set $(v_1,\cdots ,v_n)$
$fg_r$	The forged record

### Distance metric for microaggregation

The microaggregation method partitions the data records into different equivalent classes in accordance with the principle of maximum intra-class similarity and minimum inter-class similarity. Distance metric is usually used to evaluate the similarity between different records. In a relational database, a record often corresponds to an entity composed up of different type of attributes. Attributes in their own essence are used to describe the properties of a certain entity and primarily include continuous attributes and discrete attributes.

Continuous attributes are quantitative attributes as they may take on any value within a finite or infinite continuous interval. *Age*, *Height*, *Weight*, *Temperature*, etc., are all examples of continuous attributes. Discrete attributes refer to attributes with a finite number of discrete values and can be further classified into nominal attributes and ordinal attributes. The discrete nominal attributes cannot be ordered and cannot be measured and include two categories: (1) there are some semantic correlations between the discrete nominal attribute values, the taxonomy tree can be used to define the distance between those values, and (2) the discrete values of a nominal attribute have no relationships whatsoever, proximity measure can be adopted to estimate the distance between such attribute values. The discrete ordinal attribute is an attribute whose possible values have a meaningful order or ranking amongst them but the magnitude between different values is not known.

Table 4 depicts a micro data table with different kinds of attributes, wherein *Age* is a continuous attribute, *Zipcode* is a discrete nominal attribute with semantic correlations, *Sex* and *Religion* are discrete nominal attributes with non-semantic correlations,and *Capitalgain* is a discrete ordinal attribute with 3 values {moderate, good, excellent}. A series of distance metrics have been defined to assess the relations of records with multiple attributes.

#### Definition 1

(*Distance for continuous attribute [15]*). For any continuous attribute *C* in data table *T*, the distance between two values $v_i,v_j\in C$ can be defined as:

1 $$\begin{aligned} d_C(v_i,v_j)=\frac{|v_i-v_j|}{max(C)-min(C)} \end{aligned}$$

where, *max*(*C*) and *min*(*C*) refers to the maximum and minimum value of a continuous attribute *C*.

#### Definition 2

(*Distance for semantic correlation nominal attribute [26]*). For any semantic correlation nominal attribute $N_s$ in data table *T*, the distance between two values $v_i,v_j\in N_s$ can be denoted as:

2 $$\begin{aligned} d_{N_s}(v_i,v_j) =\left. {\left\{ \begin{array}{ll} 0, &{} {v_i=v_j} \\ \frac{|Parent(v_i,v_j)|}{|Tree_{N_s}|}, &{} {v_i \ne v_j} \end{array}\right. } \right. \end{aligned}$$

where $Tree_{N_s}$ refers to the taxonomy tree for semantic correlation nominal attribute $N_s$, $|Tree_{N_s}|$ is the total number of leaf nodes for $Tree_{N_s}$. $Parent(v_i,v_j)$ is the common parent node of $v_i$ and $v_j$ according to $Tree_{N_s}$, and $|Parent(v_i,v_j)|$ represents the total number of leaf nodes with the root $Parent(v_i,v_j)$.

#### Definition 3

(*Distance for non-semantic correlation nominal attribute [15]*). For any non-semantic correlation nominal attribute *N* in data table *T*, the distance between two values $v_i,v_j\in N$ can be denoted as:

3 $$\begin{aligned} d_N(v_i,v_j)=\frac{p-match(v_i,v_j)}{p} \end{aligned}$$

where *p* is the total number of non-semantic correlation nominal values exists in *N*, $match(v_i,v_j)$ is the number of matches between $v_i$ and $v_j$.

#### Definition 4

(*Distance for ordinal attribute [15]*). For any ordinal attribute *O* in data table *T*, the distance between two values $v_i,v_j\in O$ can be defined as:

4 $$\begin{aligned} d_O(v_i,v_j)=|\phi (v_i)-\phi (v_j)| \end{aligned}$$

with :

5 $$\begin{aligned} \phi (v)=\frac{rank(v)-1}{|O|-1} \end{aligned}$$

where *rank*(*v*) is the rank of value *v* in the ordinal attribute *O* in ascendant order, and |*O*| is the number of distinct values in ordinal attribute *O*.

#### Definition 5

(*Distance between two records [23]*). For a data table *T* with continuous attributes $C_i(i=1,\dots ,m)$, semantic correlation nominal attribute $N_{s_j}(j=1,\dots ,n)$, non-semantic correlation nominal attribute $N_g(g=1,\dots ,x)$ and ordinal attributes $O_h(h=1,\dots ,y)$, the distance between two records $r_1,r_2\in T$ is defined as:

6 $$\begin{aligned} \begin{aligned} d(r_1,r_2)=\frac{1}{|QIA|}&(\sum _{i=1}^{m}d_C(r_1(C_i), r_2(C_i))+ \sum _{j=1}^{n}d_{N_s}(r_1(N_{sj}), r_2(N_{sj}))+\sum _{g=1}^{x}d_N(r_1(N_g), r_2(N_g))+\sum _{h=1}^{y}d_O(r_1(O_h), r_2(O_h))) \end{aligned} \end{aligned}$$

where $d_C(r_1,r_2)$, $d_{N_s}(r_1,r_2)$, $d_N(r_1,r_2)$ and $d_O(r_1,r_2)$ are the corresponding continuous, nominal and ordinal distance functions defined in Definitions 1–4. |*QIA*| is the number of quasi-identifiers in data table *T*.

**Table 4:** A micro data table of mixed attributes.

ID	Age	Zipcode	Sex	Religion	Capitalgain
1	33	10010	F	Buddhism	good
2	35	10019	M	Catholicism	excellent
3	49	10020	F	Islam	good
4	51	10022	M	Catholicism	moderate

**Figure 1:**
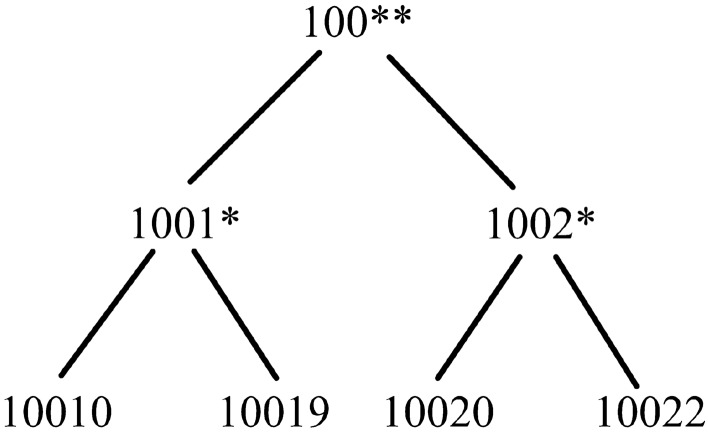
Taxonomy tree of attribute Zipcode.

#### Example 1

Let’s consider the micro data shown in Table 4. Figure 1 shows the taxonomy tree of semantic correlation nominal attribute *Zipcode*. The discrete ordinal attribute *Capitalgain* has 3 values {moderate, good, excellent}, where $rank(moderate)=1$, $rank(good)=2$ and $rank(excellent)=3$. According to Definition 5, the distance between $r_1$ and $r_2$ is $d(r_1,r_2)=\frac{1}{5} \times (\frac{|33-35|}{51-33}+\frac{2}{4}+\frac{2-0}{2}+|\frac{2-1}{3-1}-\frac{3-1}{3-1}|)=\frac{19}{45}$, and the distance between $r_2$ and $r_4$ is $d(r_2,r_4)=\frac{1}{5} \times (\frac{|35-51|}{51-33}+\frac{4}{4}+\frac{2-2}{2}+|\frac{3-1}{3-1}-\frac{1-1}{3-1}|)=\frac{26}{45}$.

### Entropy metric for microaggregation

In clustering-based microaggregation, we hope to group similar data records together to form an equivalent group during the clustering stage, while ensuring the protection of sensitive values in the equivalent group. The solution is to minimize the distance between the quasi-identifier attributes in the equivalent group and maximize the diversity of the sensitive attributes in each equivalent group. Therefore, information entropy and entropy increase have been used as the indicators of clustering for microaggregation to evaluate the degree of diversity within the equivalent group during the clustering process.

#### Definition 6

(*Entropy increase [19]*). Given an equivalent group GID, GID’ represents the equivalent group after adding a record *r*, the increase of information entropy can be defined as:

7 $$\begin{aligned} \widehat{H}(GID,GID')= H(GID)-H(GID') \end{aligned}$$

with :

8 $$\begin{aligned} H(GID)=\sum _{i=1}^{n}p_i log p_i \end{aligned}$$

where $G'=G\cup r$ is the union of equivalent group *G* with the added record *r*, *H*(*G*) is the information entropy of *G*, and $p_i$ are the probabilities of sensitive values of *G*.

#### Definition 7

(*Microaggregation metric [19]*). The microaggregation clustering metric is defined as a function to decide which record is the best choice to join the equivalent group during microaggregation clustering. The function can be be defined as:

9 $$\begin{aligned} f(GID,GID')=-\alpha \widehat{H}(GID,GID')-\beta d(\mu _{GID},r) \end{aligned}$$

where GID and GID’ represent the equivalent group before and after adding a new record *r*, *d* and $\widehat{H}$ are respectively the distance and entropy increase metrics defined in Definition 5 and 6, $\mu _{GID}$ is the centroid of the equivalent group GID. $\alpha$ and $\beta$ are the weight parameters used to adjust the proportion of the entropy increase index and the distance index, which satisfy the condition $\alpha +\beta =1$.

#### Example 2

Still take Table 4 as an example. Suppose that the first two records $r_1$ and $r_2$ have already clustered to form the equivalent group. For the rest records $r_3$ and $r_4$, which one is more suitable to join the equivalent group next? The centroid of the equivalent group is $\mu _{GID} =(34,10010,F,Buddhism,good)$. $G'=GID \cup r_3$ and $G''=GID \cup r_4$ are the new equivalent groups after adding record $r_3$ and $r_4$ respectively. According to Definition 7, there is $f(GID,G')=-0.6[-(\frac{1}{2}log\frac{1}{2}+\frac{1}{2}log\frac{1}{2})+(\frac{1}{3}log\frac{1}{3}+\frac{1}{3}log\frac{1}{3}+\frac{1}{3}log\frac{1}{3})] - 0.4[\frac{1}{5}(\frac{|34-49|}{51-33}+\frac{4}{4}+\frac{2-1}{2}+|\frac{2-1}{3-1}-\frac{2-1}{3-1}|)]= -0.081$, and $f(GID,G'')=-0.6[-(\frac{1}{2}log\frac{1}{2}+\frac{1}{2}log\frac{1}{2})+(\frac{2}{3}log\frac{1}{3}+\frac{1}{3}log\frac{1}{3})] - 0.4[\frac{1}{5}(\frac{|34-51|}{51-33}+\frac{4}{4}+\frac{2-0}{2}+|\frac{2-1}{3-1}-\frac{1-1}{3-1}|)]= -0.29$. $f(GID,G')$ is superior to $f(GID,G'')$, therefore, record $r_3$ is more suitable to join the equivalent group.

## Privacy protection microaggregation against synonymous linkage

The *K*-anonymity privacy protection method based on microaggregation avoids the generalization operation on the quasi-identifiers, and therefore, the availability of the published data is guaranteed. However, as already discussed in the Introduction, the attacker may not be able to identify the record of a targeted victim accurately but could still infer a victim’s sensitive value from the published data via the synonymous linkage between the sensitive values associated to the same equivalent group. In order to solve this problem, we propose a privacy preserving microaggregation method for handling synonymous linkage. We first design a series of indicators to evaluate the synonymous linkages between non-numerical sensitive values. Then the designed probability mass synonymous linkage was combined with the traditional microaggregation metric to form a new microaggregation metric that can mitigate synonymous attacks. While using our designed new microaggregation metric to cluster data records into equivalent groups or adjust equivalent groups when data are updated, the number of synonymous linkages can be minimized so that most of the sensitive values within the same equivalent group cannot be linked to the same generalized sensitive value.

### Predefined catalogue for the sensitive attribute Disease

This section formalizes our new method based on the privacy protection requirements of synonymous linkage. In order to facilitate the discussion, we use *Disease* as the sensitive attribute in this paper, which is a typical non-numerical attribute of semantic associations. According to human disease classifications data from Britannica (https://www.britannica.com/science/human-disease/Classifications-of-diseases), we predefined a catalogue of related diseases for the sensitive attribute *Disease* (shown in Table 5).

**Table 5:** Predefined catalogue of sensitive attribute *Disease*.

Disease			
Blood cancer	Anemia	Cardiovascular	Coronary artery
	Leukemia		Heart attack
	Lymphoma		Stroke
	Myeloma		Marfan syndrome
Lung cancer	Lung adenocarcinoma	Digestive system	Enteritis
	Lung cancer		Gastritis
	Oat-cell cancer		GERD
	Mesothelioma		Stomach flu
Brain cancer	Glioblastoma	Respiratory system	Bronchitis
	Astrocytoma		Pneumonia
	Meningioma		Emphysema
	Acoustic neuroma		COPD
Parasitic protozoan	Malaria	Skin	Acne
	Chagas disease		Pemphigus
	Sleeping sickness		Psoriasis
	Trypanosomiasis		Rosacea

### New definitions

#### Definition 8

(*The number of synonymous linkages between two values*). For any sensitive attribute SA, the number of linkage between two values $v_i,v_j\in SA$ can be defined as :

10 $$\begin{aligned} Link_{SA}(v_i,v_j) = {\left\{ \begin{array}{ll} 2, &{} {v_i=v_j} \\ 1, &{} {v_i \ne v_j\hbox { and }\gamma (v_i,v_j) \ne \emptyset }\\ 0, &{} {otherwise} \end{array}\right. } \end{aligned}$$

where $\gamma (v_i,v_j)$ is the common linked sensitive attribute value of $v_i$ and $v_j$ in the hierarchical catalogue of general common sensitive attributes.

Given *n* sensitive values $(v_1,\cdots ,v_n) \in D_{SA}$ where $D_{SA}$ denotes the domain of sensitive attribute, finding the number of synonymous linkages of between values $(v_1,\cdots ,v_n)$ comes down to determining the number of synonymous linkages for all values $(v_i,v_j)_{i\ne j} \in (v_1,\cdots ,v_n)$. So, all values $(v_i,v_j)_{i\ne j} \in (v_1,\cdots ,v_n)$ can be extend as elements of a strictly upper triangular matrix. Let $U=[(v_i,v_j)]$ such that $(v_i,v_j)=0$ for $i\ge j$ be the strictly upper triangular matrix that contains all set of values $(v_i,v_j)_{i\ne j} \in (v_1,\cdots ,v_n)$, $U=[(v_i,v_j)]$ can be fined as:

11 $$\begin{aligned} U = \begin{pmatrix} 0 &{} (v_1,v_2) &{} \cdots &{} (v_1,v_n) \\ 0 &{} 0 &{} \cdots &{} (v_2,v_n) \\ \vdots &{} \vdots &{} \ddots &{} \vdots \\ 0 &{} 0 &{} \cdots &{} 0 \end{pmatrix} \end{aligned}$$

#### Definition 9

(*Total number of synonymous linkages*). For a set of sensitive values $(v_1,\cdots ,v_n) \in D_{SA}$, the total number of linkage can be defined as :

12 $$\begin{aligned} Tlink_{SA}(v_1,\cdots ,v_n) = \sum _{i,j=1}^{n}Link_{SA}(U_{i,j}) \end{aligned}$$

where $(v_1,\cdots ,v_n)$ are the *n* sensitive values $\in D_{SA}$, $U_{i,j}$ are the strictly upper triangular matrix that contains all set of values $(v_i,v_j)_{i\ne j} \in (v_1,\cdots ,v_n)$, $Link_{SA}(U_{i,j})$ is the number of synonymous linkages between each values in $U_{i,j}$.

**Figure 2:**
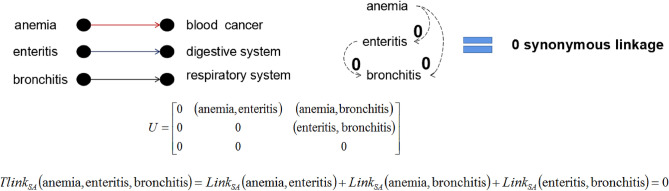
The total number of synonymous linkages of equivalent group 1 according to Table 2.

**Figure 3:**
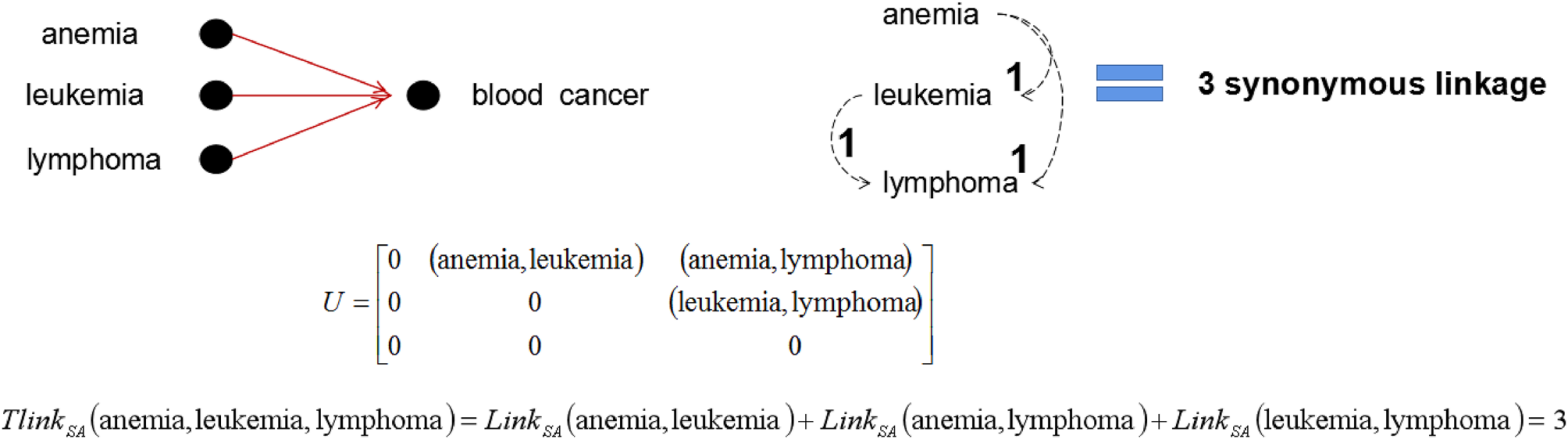
The total number of synonymous linkages of equivalent group 2 according to Table 2.

**Figure 4:**
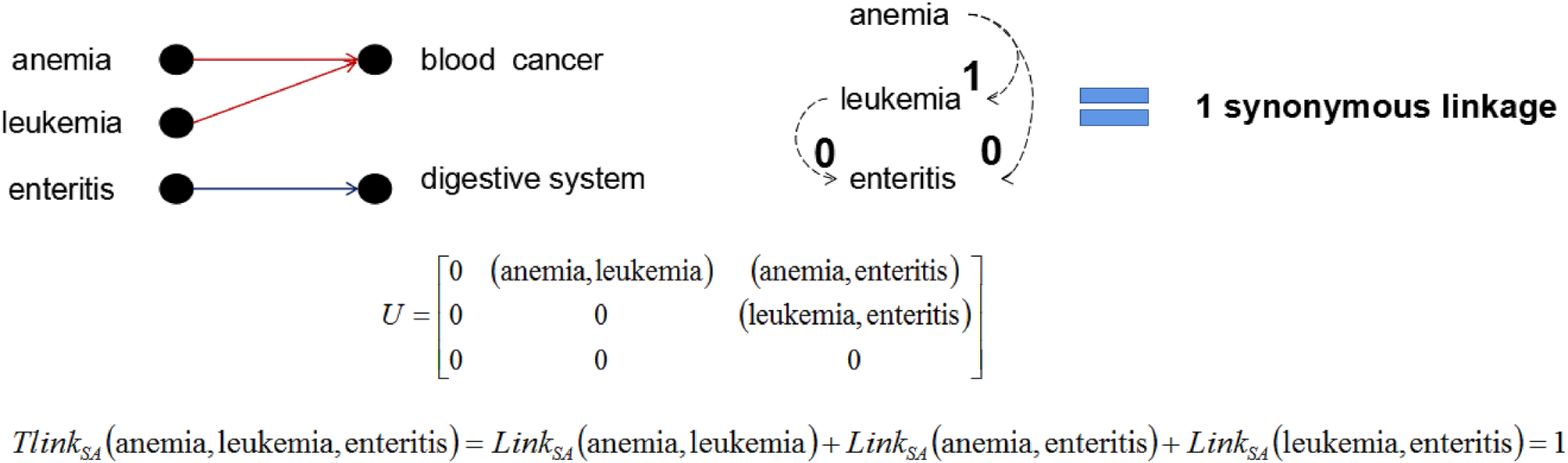
The total number of synonymous linkages of equivalent group 3 according to Table 2.

#### Example 3

Let’s considerate the previous Table 2, according to Definition 9, the total number of synonymous linkages in each equivalent group is $Tlink_{SA}(GID\_1)=0$, $Tlink_{SA}(GID\_2)=3$, and $Tlink_{SA}(GID\_3)=1$. The synonymous linkage relations of the sensitive values in each equivalent group are shown in Figs. 2, 3 and 4 respectively.

#### Definition 10

(*Probability mass synonymous linkage*). For any *n* sensitive values $(v_1,\cdots ,v_n) \in D_{SA}$, we define the probability mass synonymous linkage of set $(v_1,\cdots ,v_n)$ as :

13 $$\begin{aligned} Pr_{SA}(v_1,\cdots ,v_n)=\frac{Tlink_{SA}(v_1,\cdots ,v_n)}{n(n-1)} \end{aligned}$$

where $Tlink_{SA}(v_1,\cdots ,v_n)$ is the total number of synonymous linkages in the set $(v_1,\cdots ,v_n)$, *n* is the total number of sensitive values $\in (v_1,\cdots ,v_n)$, $n(n-1)$ is the maximum number of synonymous linkages in the set $(v_1,\cdots ,v_n)$ if all values are the same.

#### Definition 11

(*Microaggregation metric against synonymous linkage*). In order to minimize the synonymous linkage of sensitive values during the microaggregation process, we introduced the probability mass synonymous linkage on the basis of traditional microaggregation clustering metric. The new function can be defined as:

14 $$\begin{aligned} \begin{aligned} f_{link_{SA}}(GID,GID')=f(GID,GID')+\alpha Pr_{SA}(GID')=-\alpha (\widehat{H}(GID,GID')-\beta d(\mu _{GID},r)+\alpha Pr_{SA}(GID')) \end{aligned} \end{aligned}$$

where $f(GID,GID')$ is the microaggregation clustering function defined in Definitions 7, $Pr_{SA}$ is the probability mass synonymous linkage defined in Definitions 10. The functions of parameters $\alpha$ and $\beta$ are the same as those in Definition 7.

## Privacy preserving dynamic data release based on microaggregation

In order to reduce the privacy leakage caused by semantic correlation between sensitive values during data publishing, we propose a new privacy preserving publishing algorithm against synonymous linkage based on microaggregation. As an effective statistical data disclosure control technology, microaggregation can also be used to achieve anonymous protection for data publishing. Our proposed privacy preserving microaggregation algorithm adopts the classic *K*-anonymity model to prevent disclosure of individual information. Microaggregation metric against synonymous linkage (Definition 11) was adopted as a criteria to find an optimal record to join the equivalent group during data microaggregation. After microaggregation, each equivalent group should contain at least *K* records so that the individual represented by the record cannot be distinguished from the records of other ($K-1$) individuals.

The *K*-anonymity model achieves the group masking effect on quasi-identifiers by generalizing the precise value into a generalized range of value. Generalization operations will lead to the loss of information. In extreme cases, the quasi-identifier attributes of all the records are generalized to the same value range which completely destroys the value of the published data for the subsequent analysis, mining, and application. Our proposed new microaggregation metric consists of three parts. The first two parts facilitate in minimizing the distance between the quasi-identifier attributes in each equivalent group in a bid to maintain lower information loss during data microaggregation. The last part of the proposed microaggregation metric, i.e., probability mass synonymous linkage, maximizes the semantic diversity of sensitive attribute in each equivalent group. Therefore, the proposed microaggregation metric against synonymous linkage can optimize the trade-off between disclosure risk and information loss resulting from the anonymization process.

In addition, the dynamic update program is introduced into our proposed algorithm to realize the insertion, deletion, and modification of data. When some new records are required to be incorporated into the publishing data, forged records are generated and inserted into corresponding equivalent group, and further dynamic adjustment will be implemented to prevent synonymous linkage. When some records are required to be deleted, the similar dynamic adjustment process also needs to be executed to maintain the semantic diversity of the updated data. Combining the above functions (record insertion and deletion), when some records have changed and are required to be updated, we first delete the old ones form their corresponding equivalent group and then insert the new ones into the most suitable equivalent group and adjust them dynamically. The above functions ensure that our proposed privacy preserving microaggregation algorithm applicable for both the static and the dynamic data publishing scenario.

### Microaggregation publishing algorithm for the first release

Algorithm 1 describes the first publishing process of the proposed dynamic data release algorithm against synonymous linkage (DRASL). For a input data table *T* and a privacy protection parameter *K*, the first release algorithm returns the anonymous data table $T^*$ and the clustered equivalent groups *GID* according to the predefined catalogue of sensitive values. **Lines 6-22** illustrates the main process of microaggregation, where the improved microaggregation metric against synonymous linkage (Definition 11) was adopted as the criteria to find a best record to join the equivalent group during microaggregation clustering. 
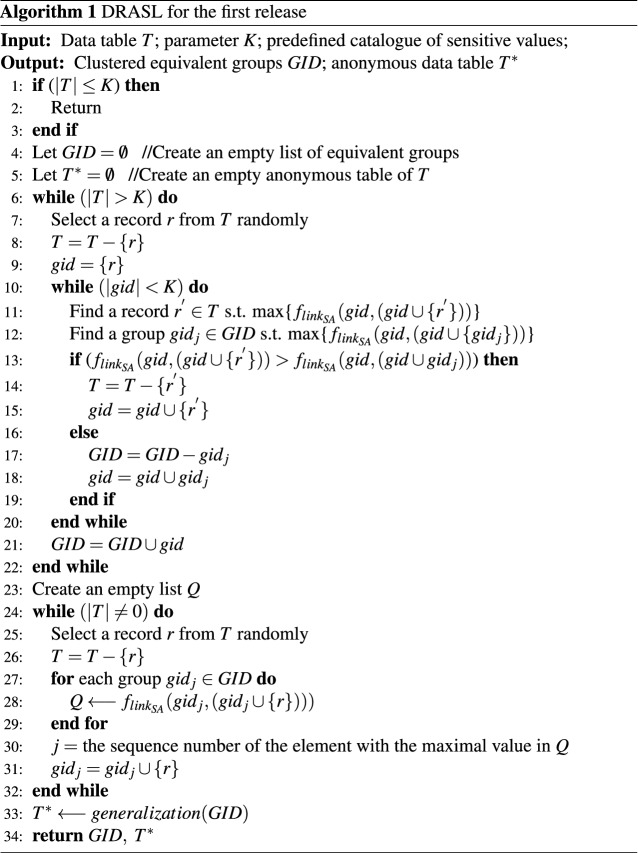


### Microaggregation dynamic adjustment for record insertion

Algorithm 2 depicts the dynamic insertion process of the proposed DRASL algorithm. The main steps were derived from the method in [20], but using the improved microaggregation metric against synonymous linkage (Definition 11) as the criteria to find a best equivalent group for the insert records.

In the case of dynamic update for record insertion, the method proposed in [20] generates forged records and insert them into equivalent groups so as to prevent sensitive information disclosure. However, there are still chances for an attacker to infer the sensitive value of an individual. **Situation 1**: for an equivalent group without forged record, when a new record *r* is added, a forged record $fg_r$ is generated randomly so that the sensitive value of $fg_r$ is different with that of *r*. However, if the sensitive value of $fg_r$ is synonymous linked with that of *r*, then, the new record is exposed to synonymous attack. **Situation 2**: for an equivalent group already with a forged record $fg_r$, when a new record *r* is added, the sensitive value of $fg_r$ will be updated into another value randomly. However, if the updated sensitive value of $fg_r$ is synonymous linked with that of the new record *r*, the new record is exposed to synonymous attack.

#### Example 4

Take the clustered equivalent groups shown in Table 6 for example, Table 7 is the updated version after a new record $$r=(23, 15032, bronchitis)$$ (shown in red) has been inserted to the equivalent group $$GID_1$$. According to situation 1, the dynamic adjustment algorithm generated a forged record $$fg_r=([21-23], [12***-15***], pneumonia)$$ (shown in blue) in the group $$GID_1$$ where the new record is belong. The sensitive values of $$fg_r$$ and *r* are different, however, by comparing the differences between Tables 6 and 7, the attacker still can conclude that the individual corresponding to the newly added record has some respiratory system disease, because “bronchitis” and “pneumonia” are synonymous linked to the respiratory system disease. The same problem may also occur in situation 2.


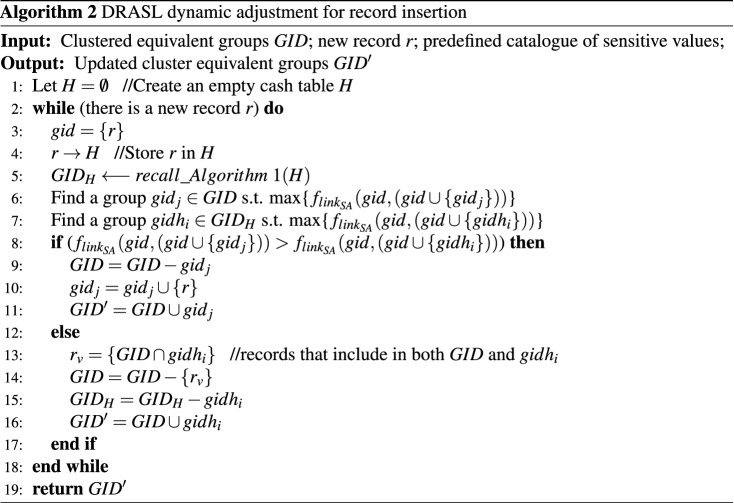

**Table 6:** Clustered equivalent groups from a micro patient table.

GID	Age	Zipcode	Disease
1	[21-22]	[12***–14***]	Enteritis
1	[21-22]	[12***–14***]	Leukemia
2	[26-28]	[18***–25***]	Bronchitis
2	[26-28]	[18***–25***]	Anemia

**Table 7:** Updated equivalent groups after new record insertion.

GID	Age	Zipcode	Disease
1	[21–23]	[12***–15***]	Enteritis
1	[21–23]	[12***–15***]	Leukemia
1	[21–23]	[12***–15***]	Bronchitis
1	[21–23]	[12***–15***]	Pneumonia
2	[26–28]	[18***–25***]	Bronchitis
2	[26–28]	[18***–25***]	Anemia

In order to solve this issue, we propose another dynamic adjustment method to protect sensitive values after Algorithm 2 (as shown in Algorithm 3). **Lines 3-4** deal with the situation that there is already a forged record $fg_r$ in the group $gid_j$. The algorithm randomly changes the sensitive value of the forged record into a new value $D_{SA}\_random\in D_{SA}$, so that the sensitive value of the forged record and the new record are different and have no synonymous linkage ($Link_{SA}(D_{SA}\_random,SA_r)=0$). **Lines 5-6** aims at the situation that there is no forged record in the group $gid_j$. The algorithm generates a new forged record $fg_r\in gid_j$ with a random sensitive value $D_{SA}\_random\in D_{SA}$ so that the sensitive value of the forged record and the new record are different and have no synonymous linkage ($Link_{SA}(D_{SA}\_random,SA_r)=0$). 
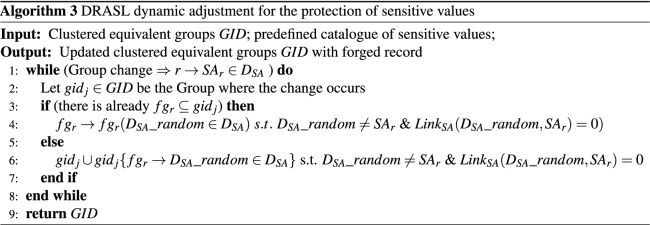


#### Example 5

Let’s consider the previous clustered equivalent groups shown in Table 6. After applying the proposed Algorithm 3, the results have changed (as shown in Table 8). Compared to previous results after new record has been added (as shown in Table 7), Algorithm 3 generated a forged record $$fg_r=([21-23], [12***-15***], acne)$$. The sensitive values of $$fg_r$$ and the new added record *r* are not only different but also not synonymous linked, because there is $$Link_{SA}(bronchitis,acne)=0$$. Therefore, the privacy protection effect on the published data has been enhanced.

**Table 8:** New record insertion after using Algorithm 3.

GID	Age	Zipcode	Disease
1	[21-23]	[12***–15***]	Enteritis
1	[21-23]	[12***–15***]	Leukemia
1	[21-23]	[12***–15***]	Bronchitis
1	[21-23]	[12***–15***]	Acne
2	[26-28]	[18***–25***]	Bronchitis
2	[26-28]	[18***–25***]	Anemia

### Microaggregation dynamic adjustment for record deletion

Algorithm 4 presents the pseudo code of the dynamic adjustment record deletion process of the proposed DRASL algorithm. In **Lines 3–5**, when the size of the equivalent group $gid_j$ after record deletion reaches or exceeds *K*, Algorithm 3 will be recalled and the deleted record *r* and equivalent group $gid_j$ will be used as the input to carry out the updating process. **Lines 6–19** are the key processes of dynamic adjustment for record deletion. When the size of the equivalent group $gid_j$ after record deletion is less than *K*, the algorithm deletes $gid_j$ from the clustered equivalent group *GID* (**Line 7**). In **Lines 9–10**, a record *r* will be randomly selected and removed from $gid_j$. Then, **Lines 11–13** evaluate which equivalent group is most suitable for the record *r* to join in according to the improved microaggregation metric defined in Definition 11. After that, the record *r* is added to the corresponding group $G_i$ (**Lines 14–15**), and Algorithm 3 will be recalled to update the clustered equivalent groups *GID* (**Lines 16–18**). 
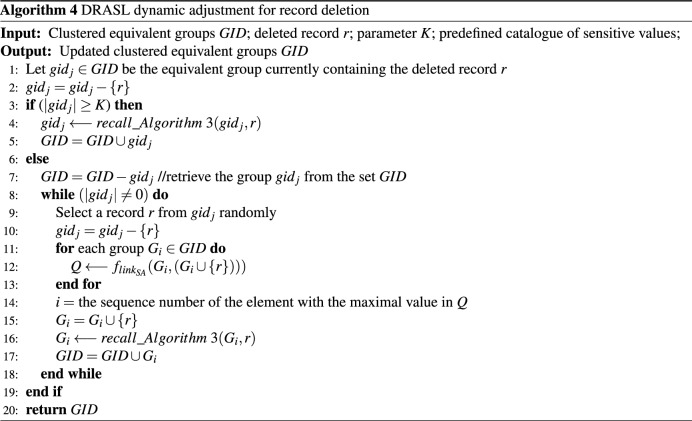


### Microaggregation dynamic adjustment for record modification

In this section, we propose the microaggregation dynamic adjustment algorithm for record modification, as shown in Algorithm 5. If the modification happened on quasi-identifiers, the old record should be deleted by calling back Algorithm 4 and the modified new record should be inserted into a most suitable equivalent group by calling back Algorithm 2 (**Lines 3–7**). If the modification happened on sensitive value, Algorithm 3 will be called back to protect the semantic diversity of the modified sensitive value (**Lines 8–15**).
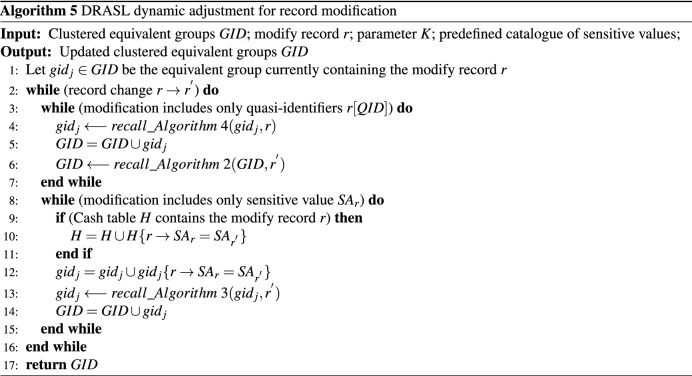


## Experimental Results

In order to evaluate the effectiveness and efficiency of the proposed algorithm, we analyze and compare the proposed DRASL algorithm with some state-of-the-art existing algorithms from the term of privacy protection effect, availability of published data, and execution time. The baseline methods include, but are not limited to, the anonymization approach based on *l*-diversity (Entropy *l*-diversity)^10^, the anonymization approach based on *t*-closeness (*t*-closeness)^11^, the one pass *K*-means algorithm (OKA)^25^, the improved *K*-anonymity algorithm based on clustering (IKA)^26^, and the data privacy protection algorithm based on microaggregation (DPP)^19^.

All the algorithms were implemented in Python and carried out on Huawei Elastic Cloud Server 8vCPUs |32GB| pl2.2xlarge.4 under Windows Server 2016 Standand 64bit for T4 with TESLA. The dataset used for the experiments were composed of seven quasi-identifier attributes and one sensitive attribute. The quasi-identifier attributes were originally selected from the Adult dataset (https://archive.ics.uci.edu/ml/datasets/Adult) obtained via the UCI machine learning repository, wherein we retained only the attributes, *Age*, *Workclass*, *Occupation*, *Education*, *Capitalgain*, *Race*, and *Gender*. The sensitive value is *Disease* which is randomly generated from 32 different diseases based on a predefined catalogue for sensitive attributes (as depicted in Table 5) and is assigned to each record in the dataset. Inaccurate records, i.e., missing values and duplicate records, were removed from the dataset.

### Privacy protection effect

Privacy preserving data publishing method based on the *K*-anonymity model primarily protects a user’s sensitive information through the group masking effect which reduces the probability of an attacker to obtain the sensitive information of a certain individual. However, there are always some correlations among the values belonging to the same type of sensitive attribute. Even if all the sensitive values in the same group are different from one another, the semantic relevance between them is inevitable. Therefore, the attacker may not be able to precisely identify the record of a targeted victim but could infer a victim’s sensitive value via the semantic relevance within the same published group. This is referred to as the synonymous linking phenomenon between the sensitive values as already discussed in this paper. The stronger the synonymous linkage is, the weaker is the group masking effect and the larger is the possibility of privacy disclosure.

In this paper, we use the total number of probability mass synonymous linkage to evaluate the privacy protection effect of sensitive attribute on the published data. Let *GID* be the final set which contains the all the equivalent groups, |*GID*| is the total number of equivalent groups, and $Pr_{SA}(SA_{G_i})$ is the probability mass synonymous linkage of equivalent group $G_i$. The total number of probability mass synonymous linkage for the set *GID* can be defined as:

15 $$\begin{aligned} Total\_Pr_{SA}(SA_{GID}) = \sum _{i=1}^{|GID|}Pr_{SA}(SA_{G_i}) \end{aligned}$$

**Figure 5:**
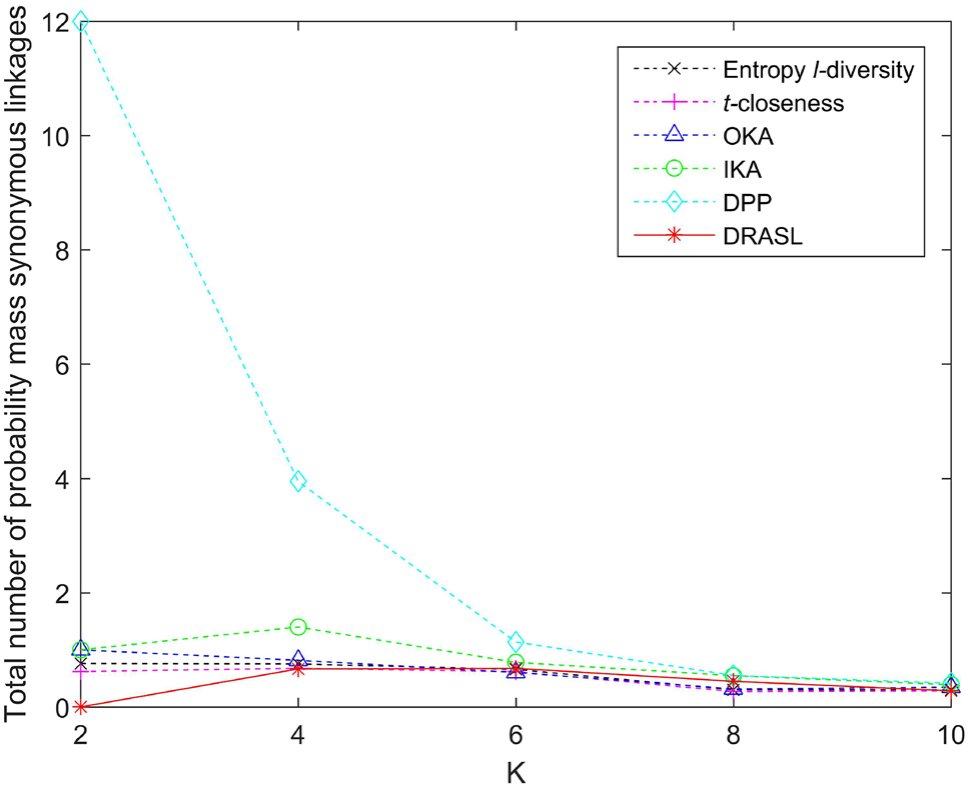
Total number of probability mass synonymous linkages for the first release.

**Figure 6:**
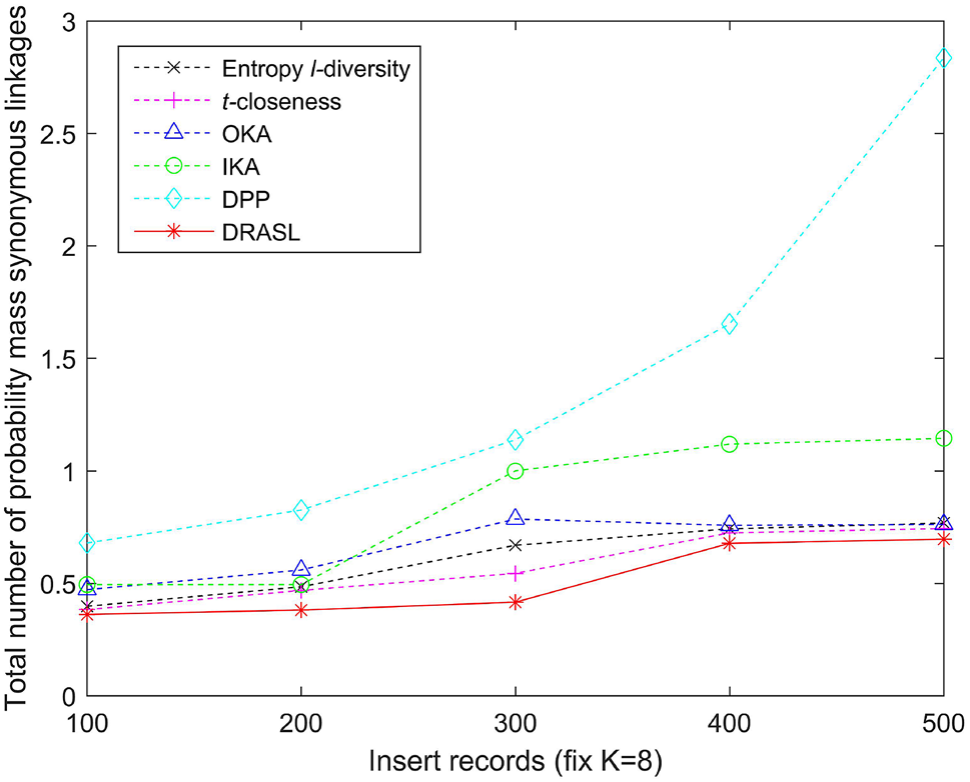
Total number of probability mass synonymous linkages for records insertion.

**Figure 7:**
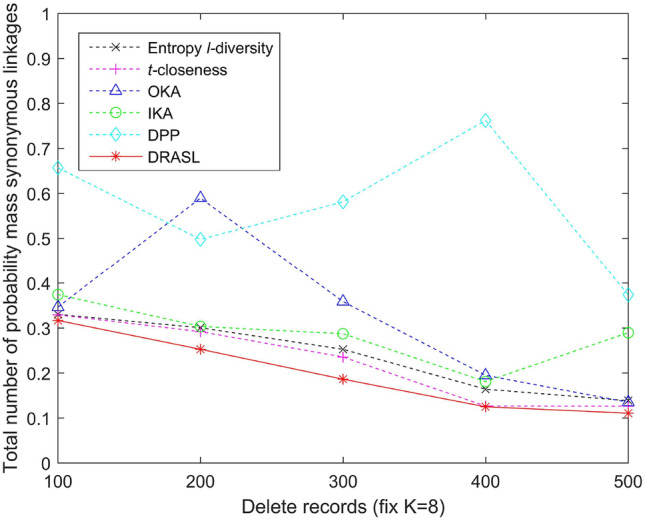
Total number of probability mass synonymous linkages for records deletion.

**Figure 8:**
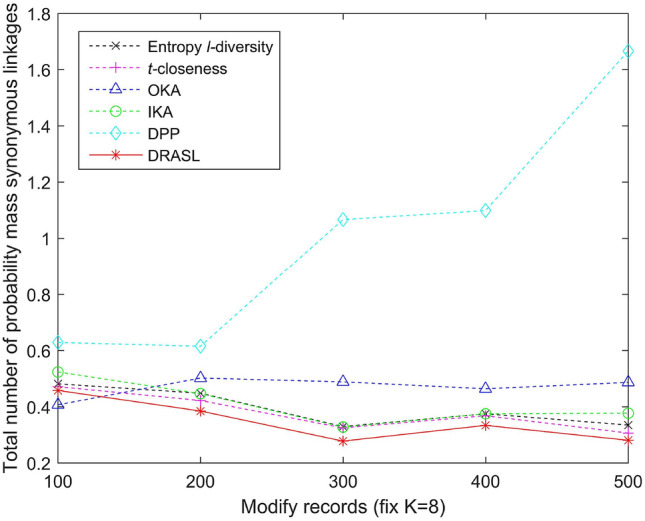
Total number of probability mass synonymous linkages for records modification.

Figures 5, 6, 7 and 8 depict the total number of probability mass synonymous linkages for all the algorithms in terms of the first release, records insertion, deletion, and modification. The weight parameters, $\alpha =0.6$ and $\beta =0.4$, are set to adjust the proportion of the entropy increase index and the distance index. The Entropy *l*-diversity method and the *t*-closeness method are typically designed for anonymous static data publishing. Therefore, the data update process of these two methods is realized by performing corresponding number of record insertion, deletion, and modification on the static dataset.

According to the definition described in Equation 13, the probability mass synonymous linkages will decrease with an increase in the total number of sensitive values within the same published group. Accordingly, we can observe from Fig. 5 that in the case of the first release, as the value of *K* increases, the total number of probability mass synonymous linkages of all the algorithms gradually decreases. In the case of dynamic update for records insertion and deletion, different numbers of records are added or deleted respectively and the size of the published group is set to be $K=8$. As the number of newly added records increases, the probability mass synonymous linkages of some of the published groups may decrease, since the increasing number of candidate records can improve the clustering effect of some groups. However, as a whole, when the published data increases and the size of the published group does not change, the number of clusters will increase as well as the total number of probability mass synonymous linkages (as depicted in Fig. 6). On the contrary, as the number of deleted records increases, both the probability mass synonymous linkages for the published groups and the total number of probability mass synonymous linkages for the entire dataset decrease (as portrayed in Fig. 7). In the case of dynamic update for records modification, half of the modifications occur on the quasi-identifiers and the other half on the sensitive values. The parameter is also set with $K=8$. The modified records bring uncertainties to the final clustering effect (i.e., the clustering effect of some published groups may have been improved, whereas, the others may have been deteriorated), and therefore, the overall status of the total number of probability mass synonymous linkages of the entire dataset has no obvious trend of change (as shown in Fig. 7).

In all of the above dynamic update situations, the proposed DRASL algorithm is superior in contrast to the other algorithms in almost all of the cases. The primary reason is that the proposed DRASL algorithm is based on the criteria of minimizing synonymous semantic linkages during the process of selecting records and adjusting the aggregation of equivalent groups. The DPP method fulfills the clustering process with the criteria of minimizing an increase in entropy. It does not consider the semantic relevance between the sensitive values at all, and therefore, performs the worst amongst all the comparison. The Entropy *l*-diversity method mandates to maintain the different values of sensitive attributes. However, it ignores the semantic relevance between them and is vulnerable to skewness attack and similarity attack. The *t*-closeness method makes the distance between the distribution of sensitive attributes in an equivalent class and the distribution of attributes in an entire data table to not exceed the threshold *t*. Nevertheless, it still cannot fundamentally prevent the synonymous linkage attack. Therefore, the proposed DRASL algorithm can provide better privacy protection for the published data from the aspect of preventing synonymous attacks.

### Availability of published data

Privacy preserving data publishing method based on the *K*-anonymity model reduces the availability of the published data to a certain extent. The primary reason is that the generalization operation carried out on the quasi-identifiers directly reduces the accuracy of the published data. The greater the degree of generalization is, the lower is the availability of the published data. In this paper, we use the average information loss to evaluate the availability of published data generated via different clustering and microaggregation algorithms.

Let *G* be an equivalent group and |*G*| be the total number of records. The amount of information loss that occurs in *G* can be defined as:

16 $$\begin{aligned} IL(G) = \frac{1}{|G|}\sum _{i=1}^{|G|}d(r_i,\mu _{G}) \end{aligned}$$

where $d(r_i,\mu _{G})$ manifests the distance between records $r_i$ and the centroid of an equivalent group *G* according to Definition 5.

Let *GID* be the set of all the equivalent groups and |*GID*| be the total number of equivalent groups in the set *GID*. The average information loss of the set *GID* is defined as:

17 $$\begin{aligned} Average\_IL(GID) = \frac{1}{|T|}\sum _{i=1}^{|GID|}IL(G_i) \end{aligned}$$

where |*T*| signifies the total number of records in the data table *T*.

**Figure 9:**
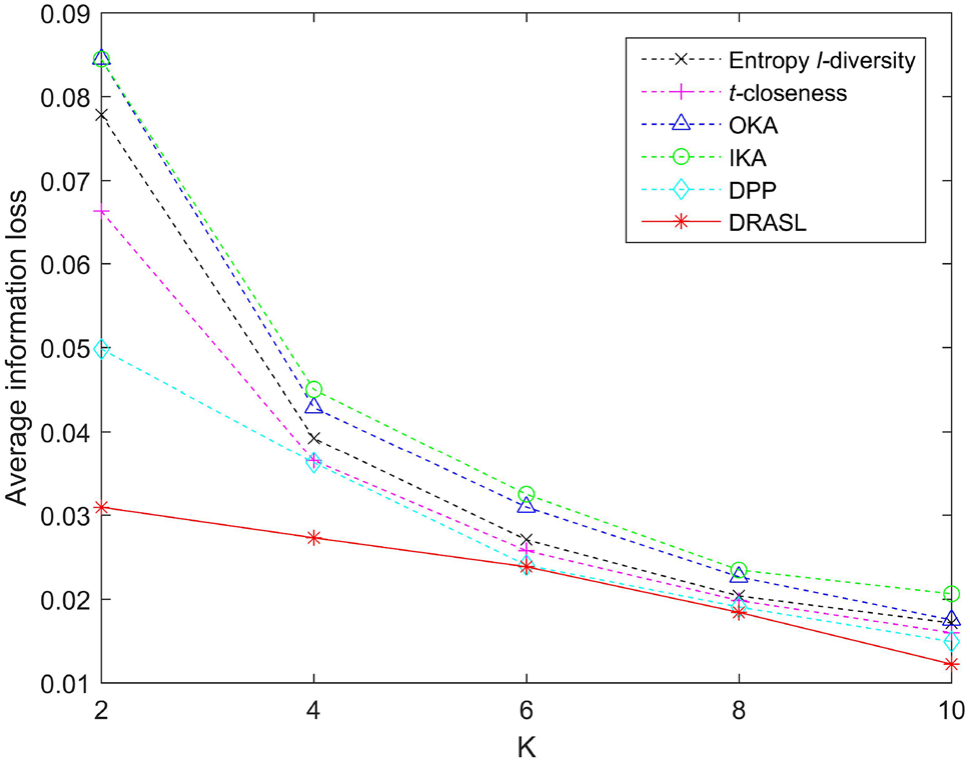
Average information loss for the first release.

**Figure 10:**
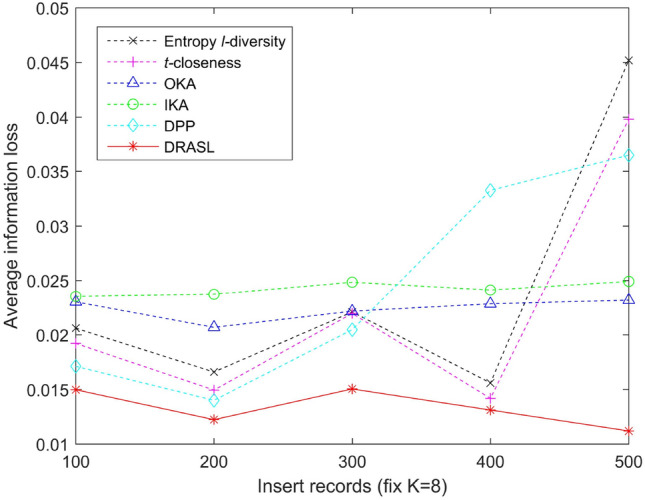
Average information loss for records insertion.

**Figure 11:**
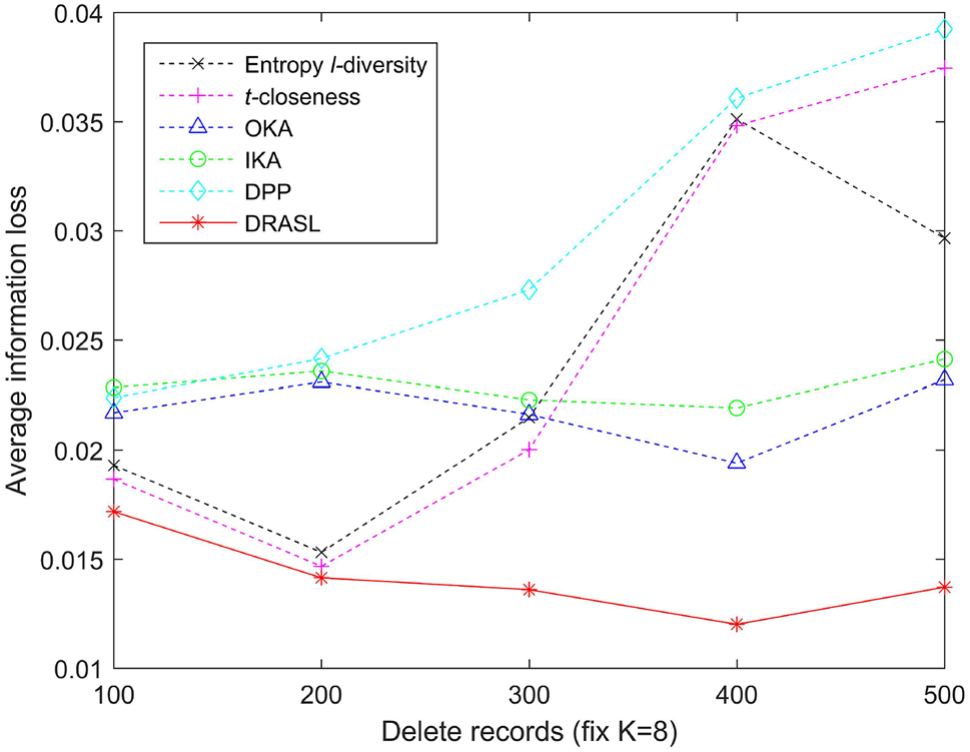
Average information loss for records deletion.

**Figure 12:**
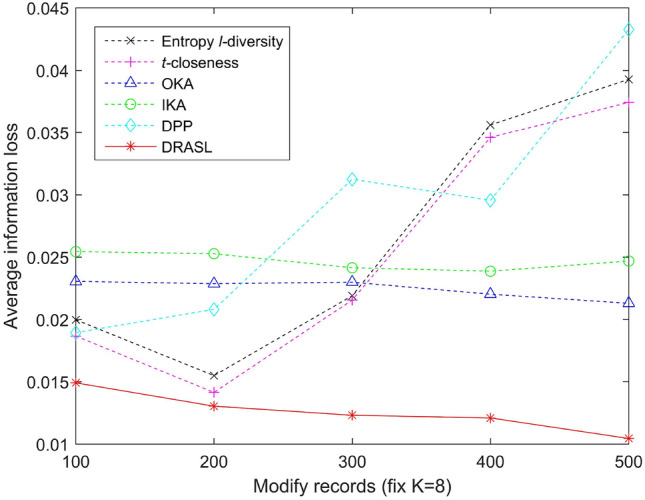
Average information loss for records modification.

Figures 9, 10, 11 and 12 portray the average information loss for all the algorithms in terms of the first release, records insertion, deletion, and modification. All the parameters and the ratio of record insertion, deletion, and modification are consistent with the previous subsection. According to the definition described in Equation 16, the amount of information loss will decrease with an increase in the total number of records within an equivalent group. Therefore, we can observe from Fig. 9 that in the case of the first release, as the value of *k* increases, the average information loss of all the algorithms gradually decreases. In the case of dynamic update of records insertion, deletion, and modification, the change in average information loss is much more complicated. Since the size of the published group is set to be $K=8$, the amount of information loss of an equivalent group varies with the sum of the distances between all the records and the centroid of the respective equivalent group, i.e., according to the Equation 16. Records insertion, deletion, or modification may change the original clustering results, thereby making the sum of distances within a single cluster to increase or decrease. Therefore, the average information loss does not show a specific trend of change (as portrayed in Figs. 10, 11 and 12). However, it is obvious that the proposed DRASL algorithm has lower average information loss in contrast to the other algorithms in all of the cases. The primary reason is that the microaggregation process of the proposed DRASL algorithm fully considers the distance between the original records (defined in Equation 14) which subsequently facilitates to minimize the impact of generalized operations and improve the availability of published data.

Both the DPP algorithm and the proposed DRASL algorithm use the insertion of forged records to realize the dynamic update and adjustment of the released data. Table 9 compares the number of forged records during the dynamic update process of the two algorithms. For the sake of fairness, the amount of records dynamically updated by the two algorithms remain the same and keep the parameter $K=8$. As noticed in Table 9, the number of forged records of the proposed DRASL algorithm are obviously less in contrast to the DPP algorithm. This also proves that the proposed DRASL algorithm introduces less interference during the process of data dynamic updating and provides better availability on the published data.

**Table 9:** Comparison of forged records after dynamic update.

Changed records	Forged records for insertion		Forged records for deletion		Forged records for modification	
	DPP	DRASL	DPP	DRASL	DPP	DRASL
100	6	3	6	5	6	5
200	6	5	6	4	6	4
300	10	7	6	3	9	4
400	16	8	6	2	9	4
500	21	6	6	2	14	4

### Comparison of execution time

**Figure 13:**
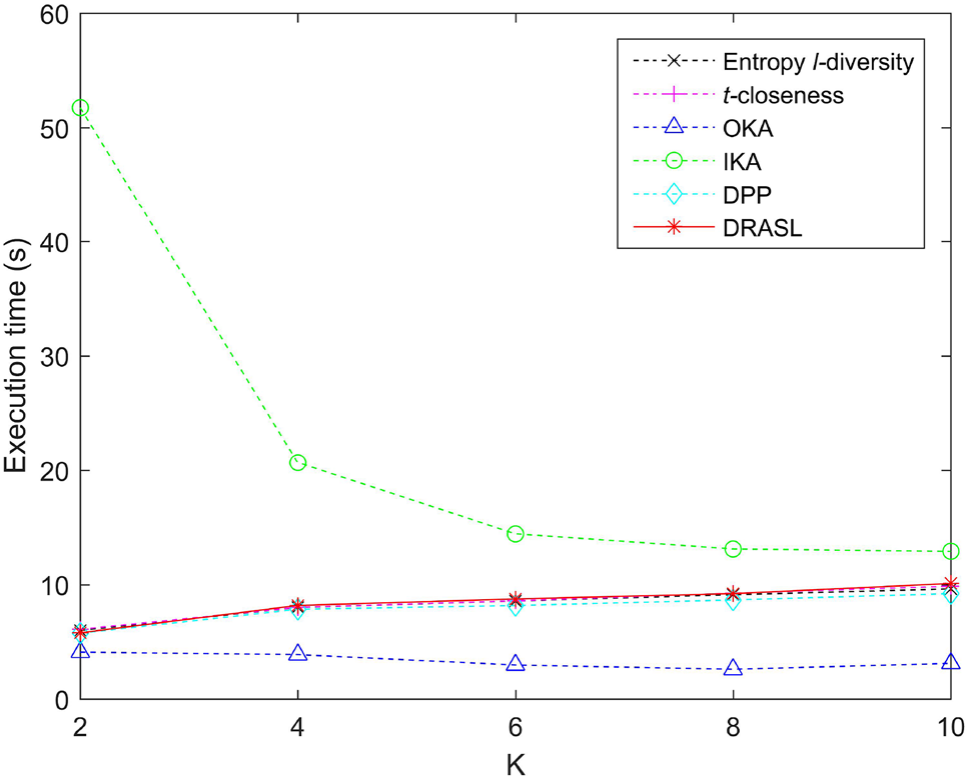
Execution time for the first release.

**Figure 14:**
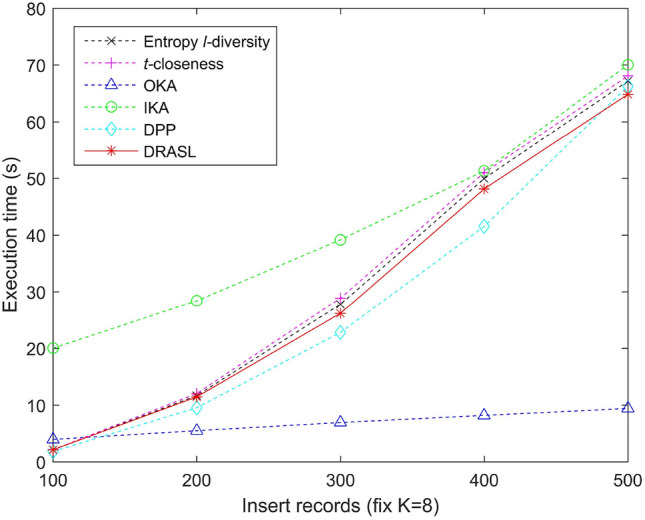
Execution time for records insertion.

**Figure 15:**
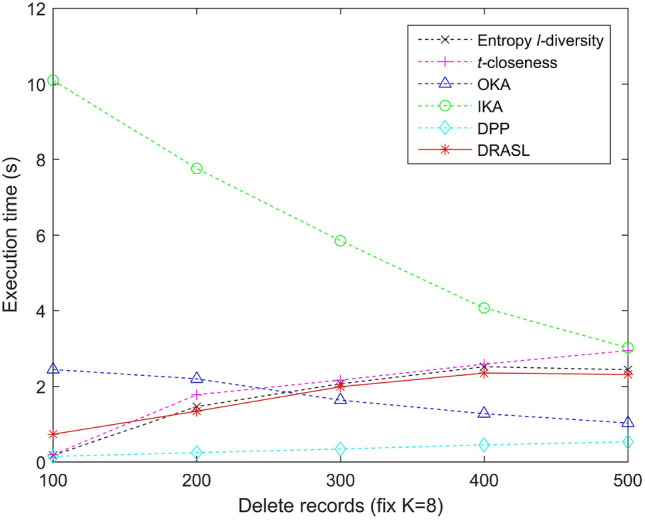
Execution time for records deletion.

**Figure 16:**
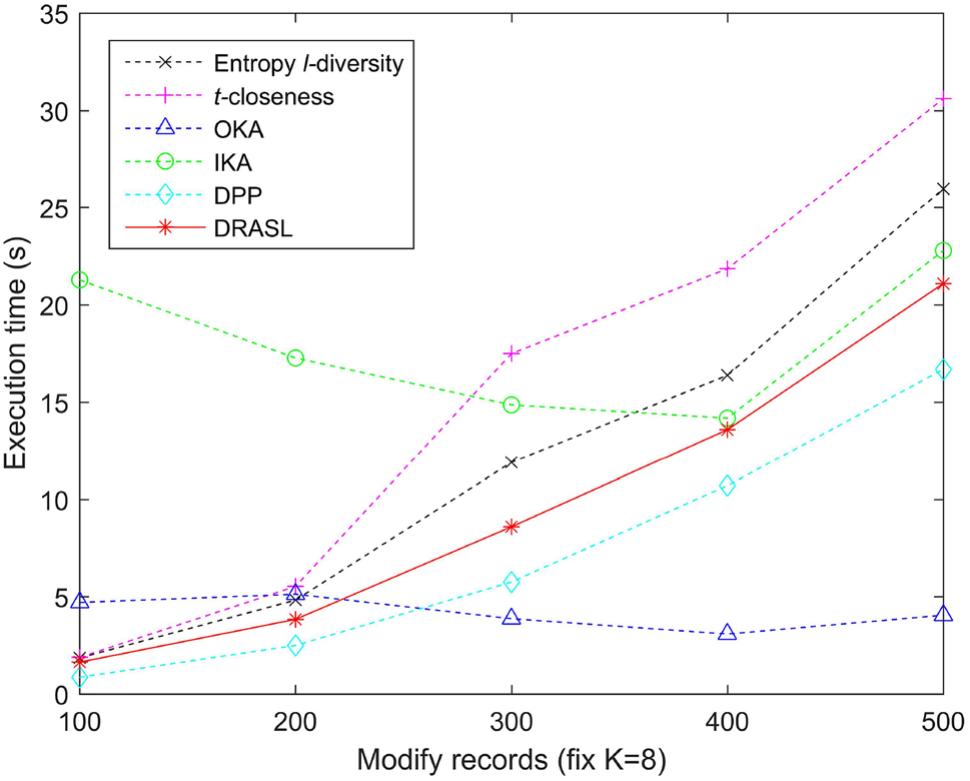
Execution time for records modification.

Figures 13, 14, 15 and 16 portray the execution time of all the algorithms in terms of the first release, records insertion, deletion, and modification. The method and proportion of record insertion, deletion, and modification remain in consistent with the previous subsection. It is pertinent to note that, all the algorithms involved in the comparison are based on clustering methods in a bid to achieve *K* anonymous privacy protection for data release. However, their specific clustering process and evaluation indicators are different from one another.

The clustering process of the Entropy *l*-diversity method, the *t*-closeness method, the DPP method, and the proposed DRASL algorithm follows a same principle, i.e., each of the equivalent group begins with a randomly selected record and continuously select the most appropriate record to join the group in accordance with different criteria. For the Entropy *l*-diversity method and the DPP method, the criteria is to minimize the increase in entropy. For the *t*-closeness method, the criteria is to keep the deviation between the distribution of sensitive attributes in an equivalence class and the distribution of attributes in an entire data table within a threshold *t*. On the contrary, our proposed DRASL algorithm takes the comprehensive effect of the distance between the records, the increase in entropy, and the number of synonymous linkages between sensitive values into consideration during the clustering process. As the clustering progresses, each equivalent group needs to incorporate records until the number of records reaches at least *K*. For a dataset with *x* records, all of the above mentioned methods have a time complexity to the order of $O(x^2)$. When the size of the published group gradually increases (as portrayed in Fig. 13) or a larger number of records in the dataset have been added/deleted/modified (as shown in Figs. 14, 15, 16), all of the above mentioned methods need to perform more adjustment operations in accordance with their clustering criteria. Therefore, the execution time for these methods demonstrates an increasing trend. In most of the cases, the execution time of the proposed DRASL algorithm is close to that of the Entropy *l*-diversity method, the *t*-closeness method, and the DPP method, and is second only to the OKA algorithm.

For a dataset with *x* records, the OKA algorithm first splits all the records into $\lceil \frac{x}{K} \rceil$ subsets. It subsequently compares the loss of information and adjust the records in each subset to achieve *K*-anonymity. Therefore, it has a time complexity of the order of $O(\frac{x^2}{K})$. The grouping and adjustment method adopted by the OKA algorithm reduces the execution time of the clustering process. Variations in the size of the published groups and the number of added/deleted/modified records have little effect on the execution time. The experimental results in Figs. 13, 14, 15 and 16 also prove this. However, the performance of the OKA algorithm in other aspects is relatively poor.

For the IKA algorithm, the calculation of the distance between the first centroid and the remaining records should be carried out for $(x-1)$ times in a bid to construct the first cluster. Subsequently, it needs $2\times (x-K-2)$ to repeat the same calculation and construct the second cluster. In order to get the third cluster, $3\times (x-2K-3)$ calculations have to be employed. Therefore, the overall time complexity is $(x-1)+2\times (x-K-2)+\dots +(\lceil \frac{x}{K} \rceil -1)\times (x-(\lceil \frac{x}{K} \rceil -2)K-(\lceil \frac{x}{K} \rceil -1)) \approx O(x^2)$. The complex iterative process of the IKA algorithm makes it relatively long to run in various situations, which is obviously in the experimental results portrayed in Figs. 13, 14, 15 and 16.

## Conclusion

The research on privacy preserving data publishing is indispensable for the further innovation and development of the promising paradigm of big data. However, data publishing methods based on *K*-anonymity model, *l*-diversity model, and their improvement strategies cannot effectively prevent the semantic linkages between the non-numerical sensitive values, thereby leading to privacy leakage problems. To address such an issue, this paper proposes a dynamic data publishing algorithm based on microaggregation. A series of indicators are designed to evaluate the synonymous linkages between the non-numerical sensitive values and to improve the clustering effect of the proposed microaggregation anonymous method. The dynamic update program is introduced into the proposed microaggregation method to realize the dynamic release and update of data. Experimental analysis suggests that the proposed method provides a better privacy protection effect and availability of published data in contrast to some state-of-the-art methods.

The privacy preserving microaggregation data publishing method proposed in this paper can be applied to a number of data publishing scenarios encompassing both numerical and categorical attributes. With the help of a distributed computing framework, the proposed method can also be run on a big data processing platform to realize a large volume of big data publishing requirements. Nevertheless, it is unable to provide privacy protection for other types of big data publishing scenarios such as unstructured data and graph data, and therefore, how to achieve privacy preserving data publishing for these types of data will be the target of our futuristic work.
